# Is voiding cystourethrogram necessary in all cases of antenatal hydronephrosis?

**DOI:** 10.4103/0970-1591.57911

**Published:** 2009

**Authors:** M. S. Ansari, Halil Suat Ayyildiz, V. R. Jayanthi

**Affiliations:** Pediatric Urology, Columbus - 432 05, OH, USA; 1 Nationwide Children Hospital, Columbus - 432 05, OH, USA

**Keywords:** Antenatal hydronephrosis, vesicoureteral reflux, voiding cystourethrogram

## Abstract

Hydronephrosis is the most common genitourinary anomaly as detected on obstetric ultrasonography and the incidence of associated vesicoureteral reflux is around 10–12%. There is inconsistency in the literature regarding which child should under go a voiding cystourethrogram (VCUG) in cases of antenatal hydronephrosis (AHN). Besides, there is a scarcity of prospective studies to demonstrate the risk of varying degree of AHN, associated reflux and their long-term impact on the kidneys. The present analysis suggests that children with AHN should undergo an ultrasound within the first month of life and further course of action should be decided on the basis of the individual case. Children with persistent moderate to severe AHN should undergo a VCUG and a functional study.

## INTRODUCTION

Antenatal hydronephrosis (AHN) is diagnosed in 1–5% of all pregnancies and the incidence of associated vesicoureteral reflux (VUR) is around 10–12%.[1–3] The significance of mild form of AHN and the need for post-natal investigation is unclear. Similarly, controversy exists regarding the natural history of incidentally detected VUR and its pathological significance.[3] Although some of the studies have shown that fetal VUR is a relatively benign condition, others suggested that children with congenital VUR may have renal damage or congenital dysplasia even before they developed urinary tract infections (UTIs).[4,5] Hence, many physicians recommend a voiding cystourethrogram (VCUG) in the early neonatal period. We try to address the question as to whether all children with AHN should undergo VCUG at a time when imaging is unquestionably improving and more mild forms of hydronephrosis are being identified.

## DISCUSSION

With the improvement in imaging techniques and the ongoing application of obstetric ultrasonography (USG) AHN is being detected more frequently. It is not clear whether this results in over testing and, subsequently, over treatment in some of these cases.[1–3]

Children diagnosed with AHN are advised a battery of tests in the early post-natal period not only to reconfirm the progress of AHN but also to diagnose the associated VUR. These tests include serial renal USG, VCUG, diuretic renogram and, occasionally, magnetic resonance urogram. There is inconsistency in the literature regarding which child should undergo VCUG in cases of AHN. Some authors recommend VCUG in children with an anterioposterior diameter ≥5 mm, while others recommend it at a higher degree of renal pelvic dilatation.[6] However, some advocate investigating every child irrespective of the grade of AHN.[7]

The primary points of debate include the post-natal outcome of mild or mild to moderate AHN, true incidence of VUR in such cases and the pathological significance of such reflux.[8] Today, we have long-term studies that have shown that both hydronephrosis and differential function improve with time in over two-thirds of the cases of AHN.[9]

The overall risk of VUR in these children is about 8.6% as compared with 1% in the normal population.[6–7] The VUR in children is associated with an increased risk of UTI. Recurrent infection and reflux may cause renal scarring hypertension, proteinuria and subsequent end-stage renal disease. That is why many authors believe that reflux should be diagnosed before the infection. Current belief is that sterile reflux is more or less harmless. Most of the mild and many moderate to severe VUR improve or resolve completely with time.[3,4]

As opposed to 10–12%, some studies have reported a higher incidence of VUR (25–33%) in these cases.[5] It is interesting to note that if VCUG is performed too early in the neonatal period, it might show a relatively higher incidence of reflux, which may not be of much clinical importance. To alleviate this predisposition, many authors believe that VCUG should ideally be delayed to 6 weeks so that we give some time for a minor degree of reflux to resolve spontaneously.[10]

There is a scarcity of prospective studies to demonstrate the risk of varying degree of AHN, associated reflux and their long-term impact on the kidneys. Many authors believe that VUR in most antenatally diagnosed hydronephrotic kidneys is physiological rather than pathological and resolves with time.[3,10] These children show a higher resolution rate of reflux without significant damage in comparison with those in whom reflux was discovered after the febrile UTI. Yeung *et al.* reported that most of the kidneys with grades I–IV VUR showed normal or near-normal differential function and morphology, i.e. no scarring on post-natal renal scintigraphy.[4] Because of the relatively benign nature of the reflux, most of the authors advocated a conservative approach in these children.

One recent metaanalysis demonstrated a significant risk of associated pathology (VUR) in both mild and moderate AHN (11.9 and 45.1%, respectively) thus potentially warranting more additional tests such as VCUG and renal dynamic scan.[7] But, one should be careful in interpreting these data as the studies analyzed did not use the same ultrasound criteria for pre-natal and post-natal imaging protocols to define AHN. The timings of antenatal USG as well as post-natal VCUG were not uniform. Likewise, some of the authors reported a prevalence of a higher grade of VUR (≥III, up to 80%) in association to AHN. But, the authors reported a 50% chance of resolution even in the presence of high-grade reflux (grades III–V) by the age of 16 months and further predicted a higher improvement rate with increasing age.[3]

USG cannot be a substitute for VCUG but, if it is carried out meticulously, the chances of missing a significant pathology are less likely. The negative predictive value of a normal post-natal ultrasound has been reported to be as good as 98.9%.[10] Children with AHN should undergo an ultrasound within the first month of life and further course of action should be decided on the basis of the individual case. Children with persistent moderate to severe AHN should undergo a VCUG and a functional study.[7]

Further prospective work in this area is definitely needed. Until then, there should be an individualized approach depending on the merit of the case. Before ordering a VCUG in cases of AHN, the physician has to really look into its objectives and achievements, i.e. risk benefit ratio, burden on health care, cost effectiveness and unwanted parent anxiety.
